# Organotypic Cultures as a Model of Parkinson´s Disease. A Twist to an Old Model

**DOI:** 10.1100/tsw.2009.68

**Published:** 2009-08-11

**Authors:** Katja Stahl, øivind Skare, Reidun Torp

**Affiliations:** ^1^ Centre for Molecular Biology and Neuroscience, and Department of Anatomy, Institute of Basic Medical Sciences, University of Oslo, Norway; ^2^ Norwegian Institute of Public Health, Department of Genes and Environment, Division of Epidemiology, Oslo, Norway; ^3^ Department of Public Health and Primary Health Care, University of Bergen, Norway

**Keywords:** Parkinson’s disease, organotypic cultures, 6-hydroxydopamine (6-OHDA), confocal microscopy

## Abstract

Organotypic cultures from the ventral mesencephalon (VM) are widely used to model Parkinson's disease (PD). In this method, neurotoxic compounds have traditionally been applied to the media to induce a uniform dopaminergic (DAergic) cell death in the tissue slices, regardless of the variation existing among slices. This study demonstrates a refinement of the toxic induction technique. We show that unilateral application of 6-hydroxydopamine (6-OHDA) at the tissue surface by means of a microelectrode causes a precisely localized cell death that closely resembles an *in vivo* stereotactic model. This technique introduces an internal control that accounts for variation between slices and enables a precise quantification of the cell loss due to the toxin in use. We characterized organotypic VM cultures in terms of effects of 6-OHDA toxicity and number of DAergic neurons as judged by immunofluorescence and Western blots. Our findings illustrate that this new application technique greatly improves the representativeness of organotypic cultures as a model for PD.We characterized organotypic VM cultures in terms of effects of 6-OHDA toxicity and number of DAergic neurons as judged by immunofluorescence and Western blots. Our findings illustrate that this new application technique greatly improves the representativeness of organotypic cultures as a model for PD.

### INTRODUCTION

Parkinson's disease (PD) is a progressive neurodegenerative disease characterized by a selective loss of dopaminergic (DAergic) cells in the substantia nigra pars compacta (SNpc) in the midbrain and a subsequent deficiency of striatal dopamine (DA). As the disease does not occur spontaneously in animals, considerable efforts have been devoted to the development of models that reliably mimic the pathological features of the disease. DA-selective neurotoxic compounds, such as 1-methyl-4-phenyl-1,2,3,6-tetrahydropyridine (MPTP), 6-hydroxydopamine (6-OHDA), and rotenone, have been widely used to model PD, as they induce rapid DAergic cell death and subsequent motor deficits in both rodents and primates[1,2,3,4].

*In vivo*, such neurotoxins are stereotactically injected into the SN or striatum in one hemisphere to induce unilateral DAergic cell death[5,6,7,8]. These models convey high representativeness, as they maintain the whole biological system intact along with all systemic parameters. However, the complexity limits well-controlled experimental manipulations. *In vivo* models are also time consuming and implicate suffering of the animals involved.

Organotypic cultures represent an *in vitro* model that maintains the *in vivo* cellular interactions and three-dimensional structure of a host region[9], and thus allows one to perform manipulations that are more flexible than those *in vivo*. Moreover, the method generates several repetitions from each animal and thus greatly reduces the number of animals required. The relative preservation of nervous tissue is achieved by culturing tissue slices at an air/liquid interface, either by continuously rotating the preparation to provide cycles of feeding and oxygenation periods[10], or cultivating them on semiporous membranes[11].

Applications of toxic compounds to organotypic cultures from the ventral mesencephalon (VM) have been used to model PD. Traditionally, the toxins have been applied directly in the medium[12,13,14,15]. This approach induces rapid DAergic degeneration with equal distribution both within and between tissue slices resting on the same membrane. Thus, this technique generates uniform DAergic cell death among exposed tissue, but does not account for natural variation that occurs among slices, such as differences in the number of DAergic cells along the rostrocaudal axis. Due to lack of internal controls, such variation might bias the definite amount of DAergic cell death and thus create false positives.

In the present study, we have refined the technique for induction of DAergic cell death in organotypic cultures so as to resemble *in vivo* models more reliably and thus increase the representativeness of the model. By means of a unilateral toxic application technique in which the toxin is applied at the tissue surface at the main DAergic cell groups in one hemisphere of the VM, namely the A8 DAergic cell group in the retrorubral field (RRF), A9 in the SNpc, and A10 in the ventral tegmental area (VTA), we demonstrate a method for precise quantification of the DAergic cell number in the two hemispheres of the same slice. The method thus provides an internal control that enables a direct comparison to the toxic treatment and thus eliminates natural variation as a confounding factor. We have used the unilateral application technique to reveal clear advantages in regard to quantification precision and *in vivo* similarity as compared to the previous toxic bath model. All together, the refined technique denotes increased representativeness of organotypic cultures as a model for PD.

### MATERIALS AND METHODS

The method has been videotaped and can be viewed by clicking on the image below or this link.

#### Animals and Preparation of Tissue

Postnatal (P5) Wistar rats (Scanbur BK AS, Nittedal, Norway) of mixed gender were used as donor animals. All animals were treated in accordance with the European Convention (ETS 123 of 1986), and conformed to National Institutes of Health guidelines for the care and use of laboratory animals. The rat pups were anesthetized by inhalation of Isofluran (Baxter Healthcare, Deerfield, IL) before cervical decapitation. The brain was quickly removed and the VM was isolated by dry dissection. The tissue was then cut in a transverse plane at 400 µm using a tissue chopper (McIlwain Tissue Chopper, The Micle Laboratory, Cambridge, U.K.). The chopped tissue was placed in glucose-enriched, ice-cold Gay's balanced salt solution (GBSS) (1.5 m*M* $CaCl_2$, 5 m*M* KCl, 0.2 m*M* $KH_2PO_4$, 1 m*M* $MgCl_2$, 0.3 m*M* $MgSO_4$, 137 m*M* NaCl, 2.7 m*M* $NaHCO_3$, 1 m*M* $NaH_2PO_4$, and 5.6 m*M* glucose) with 27.8 m*M* glucose (Braun, Melsungen, Germany) added before use, and the slices were separated carefully. Only intact slices were included; far caudal and rostral slices were discarded. All operations were performed under sterile conditions.

[redacted]

Culture media was purchased from Invitrogen Life Science, Carlsbad, CA. All other chemicals were from Sigma-Aldrich, St. Louis, MO, unless otherwise specified.

### Organotypic Cultures

Each slice of tissue was transferred to semiporous membranes (pore size 1 µm) mounted in plastic inserts (BD Falcon Cell Culture Inserts, VWR International, West Chester, PA). Two or three explants from the same animal were placed on each membrane and transferred to six-well culture trays (Techno Plastic Products Ltd, Zurich, Switzerland), in which each well contained 1 ml of Stoppini medium composed of 50% MEM with Earl's salts without L-glutamine, 25% Hank's balanced salt solution (HBSS), 25% inactivated calf serum (PAA Laboratories, Pasching, Austria), 50 U/ml Penicillin G, 50 µg/ml streptomycin, and 1 m*M* L-glutamine (Cambrex, Charles City, IA). Before use, 36 m*M* glucose was added to the medium. The culture trays were incubated at 36ºC with 5% $CO_2$ in atmospheric air (Heto-Holten $CO_2$ incubator, Heto-Holten, Allerod, Denmark). The medium was replaced after 5 days *in vitro* (DIV). The tissue was cultivated in organotypic cultures for 8 days in total.

### Two Techniques for Application of 6-Hydroxydopamine

Toxic compounds have traditionally been applied in the cell culture media to induce DAergic cell death. In this study, cultures were exposed to 6-OHDA unilaterally at the tissue surface at DIV2. 6-OHDA with ascorbic acid was dissolved in sterile salt water (0.9% NaCl in $dH_2O$) into a concentration of 1000 or 200 µ*M*. Glass electrodes (borosilicate glass capillaries, outer diameter 1.5 mm, inner diameter 0.86 mm, Clark Electromedical Instruments, Pangbourne, U.K.) with an apex diameter of approximately 2 µm were pulled by a patch pipette puller (PP-830, Narichige, Japan), and the electrodes were filled with 6-OHDA solution and connected to a Picospritzer II (General Valve Corporation, Fairfield, NJ). Depending on the electrode, the apparatus was adjusted to 3–9 msec puff with 1–2 psi air pressure. Holding the electrode 1–2 mm from the tissue surface to avoid physical contact and possible tissue injury, the toxic solution was applied at the tissue surface. The application was performed at three sites in one hemisphere; one drop in the VTA (A10), one in the SNpc (A9), and one in the RRF (A8) (Fig. 1). The drop was ensured to be absorbed by the tissue to keep the site of exposure under control. The toxic treatment was performed at room temperature and 6-OHDA was kept on ice protected from light. The wells were placed in glucose-enriched Stoppini medium and were transferred back to their original medium immediately after the exposure, leaving the tissue out of the incubator for maximally 10 min.

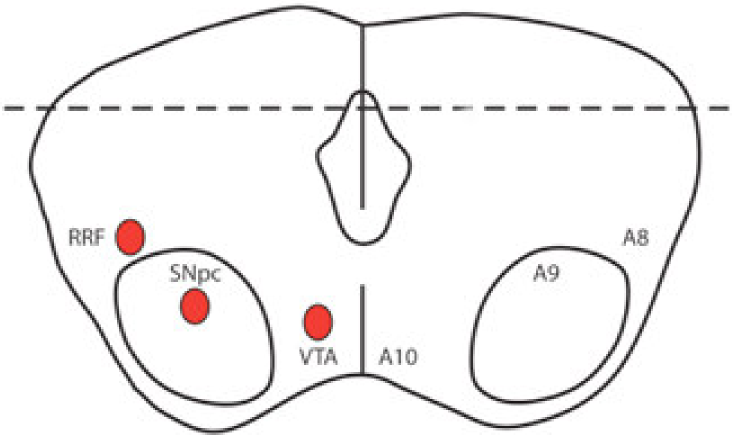

**FIGURE 1.** Illustration of exposure sites for 6-OHDA in the VM. Horizontal line illustrates removed dorsal area. Red circles correspond to exposure sites in the main DAergic cell groups localized in RRF (A8), SNpc (A9), and VTA (A10).

In addition to the unilateral exposure method, we applied high (1000 µ*M*) or low (200 µ*M*) concentrations of 6-OHDA in the culture media to be able to compare our method to the previously used toxic bath model. The slices were incubated together with the toxin-enriched medium for 6DIV.

### Tissue Homogenization and Western Blotting

Ventral midbrain homogenates in 0.32 *M* sucrose, 50 m*M* EDTA, 2 m*M* HEPES, and protease inhibitors (Roche Diagnostics, Mannheim, Germany) were centrifuged for 5 min at 3500 rpm at 4°C. The supernatant was collected and centrifuged for 30 min at 17,000 rpm at 4°C to collect water-soluble proteins. 10% Criterion Gels (Bio-Rad Laboratories, Hercules, CA) were loaded with 0.5 µg/µl protein solution, and SDS electrophoresis and blotting were performed using the Bio-Rad Criterion System and PVDF membrane (Bio-Rad Laboratories).

The membranes were blocked in 5% skimmed milk in 0.1 *M* Tween 20 (TBS-T) for 30 min on incubation over night with primary polyclonal antibodies from rabbit against tyrosine hydroxylase (TH-ab) (1:500, Chemicon, Billerica, MA) and monoclonal antibodies against β-actin (1:1000, Abcam, Cambridge, MA) diluted in blocking solution. The membranes were rinsed in TBS-T before incubation with antirabbit IgG (γ-chain specific)-alkaline phosphatase produced in mouse (1:1000, Sigma-Aldrich) or antimouse IgG (whole molecule) $F(ab')_2$ fragment alkaline phosphatase produced in sheep (1:1000, Sigma-Aldrich) in TBS-T for 1 h. The incubation was followed by extensive washing. The blots were developed using ECF substrate (GE Healthcare, Uppsala, Sweden) and then visualized with a Typhoon scanner (Typhoon 9410, Variable Mode Imager, Amersham Biosciences, Fairfield, CT).

### Immunofluorescence and Confocal Microscopy

The tissue was fixated in 4% paraformaldehyde (PFA) in 0.1 *M* natrium phosphate (NaPi) over night at DIV8. Following washing in 0.01 *M* phosphate buffered saline (PBS, pH 7.4), the tissue was blocked in blocking solution containing 10% normal calf serum (NCS), 1% bovine serum albumin (BSA), and 1% Triton-X-100 in PBS for 1 h. Sections were subsequently incubated with polyclonal antibodies from rabbit against TH (1:1000, Chemicon) or monoclonal mouse anti-TH (1:1000, Chemicon) in 3% NCS, 1% BSA, 1% Triton-X-100, and 0.05% sodium azide in PBS over night at room temperature. Following washing, sections were incubated in Cy3 donkey antirabbit or Cy3 donkey antimouse (1:1000, Jackson ImmunoResearch Laboratories, West Grove, PA) in a 3% NCS, 1% BSA, and 1% Triton X-100 in PBS for 1 h. Sections were again rinsed, mounted with Prolong Gold Antifade Reagent with 4',6-diamidino-2-phenylindole (DAPI) (Invitrogen Life Science), and coverslipped.

Confocal images were collected with a LSM 5 Pascal Confocal Microscope (Zeiss, Oberkochen, Germany) using both a 20× and 40× objective. Cy3 fluorescence was captured at 568 nm.

### Statistical Analysis

In order to determine the difference between the two hemispheres quantitatively, the number of TH-immunoreactive cells (TH-ir) was counted in the two hemispheres of the same slice. For every hemisphere, two 20×-magnification images were collected from the SN, and the total number of TH-ir cells was summarized as one observation and compared to its counterpart. The images were collected at parallel sites from each side. The criteria for the TH-ir cells included in the analysis were (1) a clearly expressed nucleus, (2) an intact cytoplasm, and (3) a soma size larger than 10 μm. Finally, to compensate for the cells lining the border, (4) cells on the left and upper border of the image were included, whereas cells on the right and lower border were discarded.

In the statistical analysis, we have count data $Y_{wrs}$ for well $w$, replicate $r$, and side $s$. For each replicate, the observations come in pairs, one observation for each side, where one of the sides is treated with a toxin. The expected effect of a toxin is a lower cell count. The counts were assumed to be Poisson distributed and we used a Poisson mixed model:

$$Y_{wrs} \sim \text{Poisson}\ (\lambda_{\text{wrs}}) \qquad (1)$$

$$\log(\lambda_{\text{wrs}}) = \mu + T_{wrs}\alpha + u_w + v_{wr} \qquad (2)$$

where $T_{wrs}$ is 1 if the side is induced by a toxin, and 0 otherwise. The terms μ and α are fixed effects. To adjust for the dependency in measurements, random effects were added for wells ($u_w$) and replicates ($v_{wr}$), where replicates were nested within wells. The random effects are normally distributed random variables. The term $\lambda_{\text{wrs}}$ is the intensity of the Poisson distribution and is the expected count in an area of unit size.

The fixed and random effects were estimated by maximized likelihood using the R function glmer in the lme4 package.

## RESULTS

### Identifying Natural Variation among Slices

In order to demonstrate how natural variation among tissue slices might bias the effect of toxic compounds, we identified the irregular distribution of DAergic cells along the rostrocaudal axis (Fig. 2A–

H). Compared to medial slices that contain densely packed, large-size, TH-ir cells (Fig. 2B–F), caudal and rostral slices are characterized by a more sparse distribution of smaller TH-ir cells, indicating that the SN is most pronounced in medial slices of the axis. Far caudal and rostral slices are rich in fibers, as would be expected due to their projections in the striatum, and do not clearly express TH-ir cells (Fig. 2A and H). In addition to axonal differences, variation can occur as a result of the experimental procedure, such as asymmetrical chopping and a different degree of survival among wells. These facts imply that natural variation accounts for perceptible dissimilarities among slices, which increase the need for internal controls.

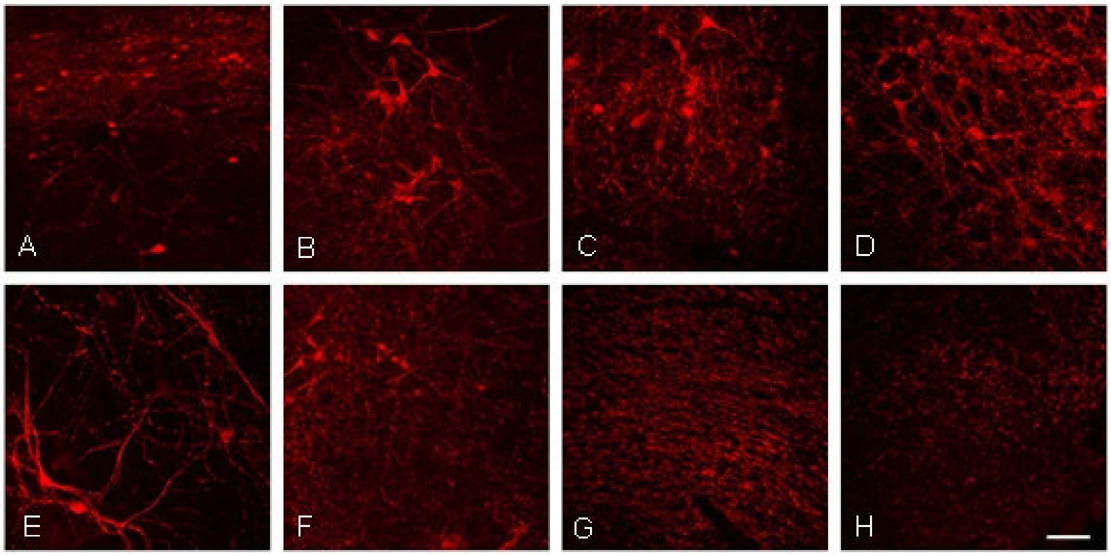

**FIGURE 2.** TH-ir cells vary along the rostrocaudal axis of SN (A–H). The TH-ir cells and fibers differ in number and size throughout the axis. Far rostral (A) and far caudal (H) slices contain smaller and sparsely distributed TH-ir cells compared to more medial slices (B–F). Further, far rostral and caudal slices show a fiber-rich appearance compared to medial slices (G). Scale bar; 50 μm.

### Toxic Application

In the refined method where 6-OHDA (200 or 1000 μm) was unilaterally applied at the tissue surface, we found that DAergic cell death was restricted to the hemisphere exposed to the toxic compound, whereas the untreated hemisphere remained intact. A clear distinction in the number of TH-ir cells along the midline of individual cultures indicated that the toxin exerted its effect locally, without diffusion to the opposite hemisphere. The Western blotting of TH confirmed the immunocytochemical results (Fig. 3A and B).

The clear difference between the two hemispheres was further confirmed statistically by showing highly significant effects of the toxin on TH-ir cell counts (Table 1). Transforming the estimates back to the normal scale, we get an estimated median cell count of 14.45 for control side and 2.84 for the toxin-injected side. This corresponds to a cell reduction of approximately 80% (Fig. 4).

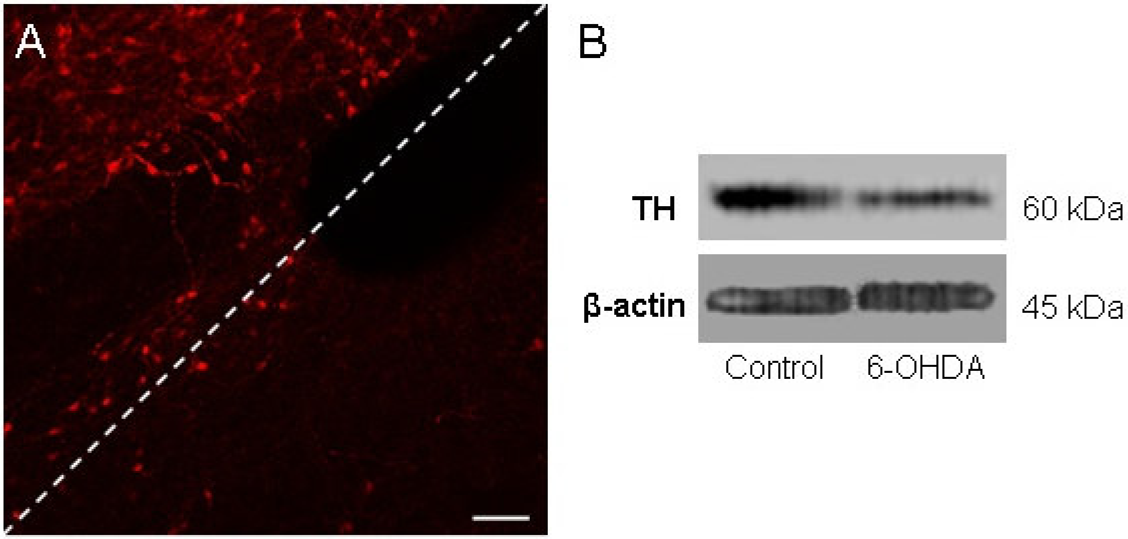

**FIGURE 3.** Both immunocytofluorescence and Western blotting showed that unilateral application of 6-OHDA results in local degeneration of TH-ir cells. The dashed midline in panel A indicates clear separation between the two hemispheres in regard to the number of TH-ir cells. The Western blot (B) confirms the degeneration in the affected hemisphere by showing a weaker band of TH at 60 kDa, while the β-actin band at 45 kDa remains stable. Scale bar; 100 µm.

**TABLE 1**
**Count Data: Estimates and *p* Values**

| Side | Estimate (Log Scale) | *p* Value |
|---|---|---|
| Control | 2.67 | $< 2 * 10^{-16}$ |
| Toxin vs. control | –1.62 | $< 2 * 10^{-16}$ |

The estimates for the variances between wells were estimated to be zero, while the variance between replications was estimated to be $0.645^2$ on the log scale. On the normal scale, this variation corresponds to a standard deviation of approximately 12.76 counts for the control hemispheres and 2.50 for the toxin-induced hemisphere. In addition to this variation between replicates, we have the Poisson residual variation.

High concentrations of 6-OHDA (1000 µ*M*) resulted in complete cell death at the exposed hemisphere (Fig. 5C), whereas low concentrations (200 µ*M*) spared approximately 20% of the cells (Fig. 5A). For toxic bath cultures, we found that the toxin generated substantial cell death as compared to slices not exposed to the toxin (Fig. 5E–H).

### DISCUSSION

Organotypic cultures grown by the interface method retain a three-dimensional cytoarchitecture that closely resembles the original tissue. In addition, this approach allows *in vitro* manipulations and full control over the experimental factors. The preserved neuron-glial interaction provides a microenvironment that facilitates cellular differentiation and enables long-term survival of neurons[16]. Thus, the method is ideally suited for studies of chronically applied drugs, protection against toxic agents, and regeneration of neuronal pathways, all of which are important in the development of a cytoprotective treatment for PD.

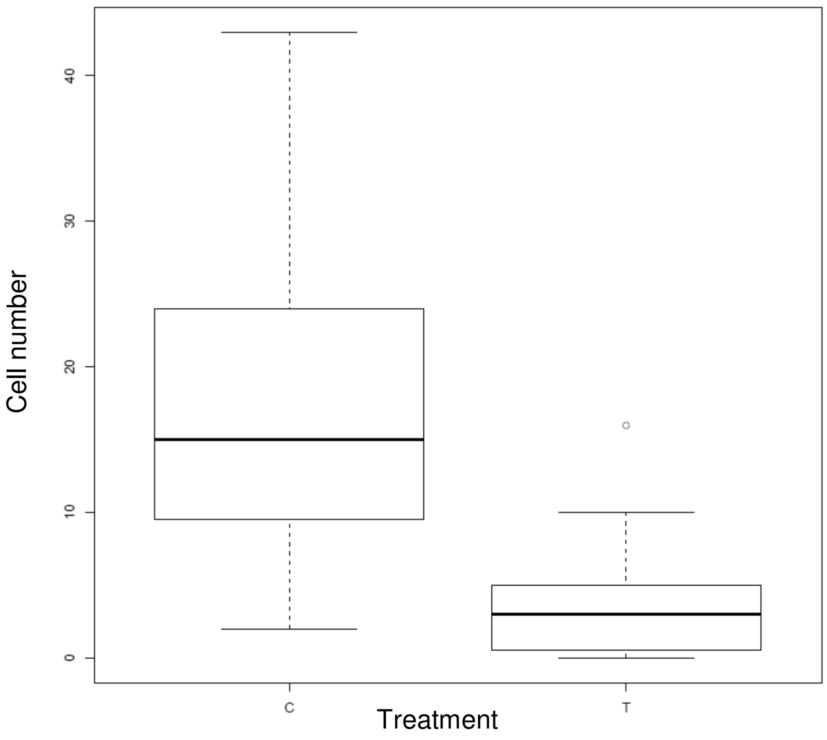

**FIGURE 4.** The box plot illustrates the difference in TH-ir cell number in the hemispheres exposed to 6-OHDA (T) and control hemispheres (C), displaying a highly significant difference between the two hemispheres ($p < 2 * 10^{-16}$). Boxes extend from the 25$^{th}$ percentile to the 75$^{th}$ percentile, with a horizontal line at the median; 14.45 TH-ir cells for control side and 2.84 for the toxin side. Whiskers represent maximum and minimum values, excluding outliers (circles). Abbreviations: C, control; T, toxin.

Using early postnatal rats as donor animals, the cytoarchitectural organization more closely resembles that of adult rats, offering an obvious benefit when modeling age-related diseases, such as PD. Moreover, when studying cytoprotective mechanisms subsequent to toxic application, tissue from prenatal animals might confound the effect by natural mechanisms that promote cell survival, such as high endogenous levels of neurotrophic factors. An immense problem that rises, however, is axotomy. Already at embryonic day 17 (E17), afferent DAergic fibers start their projections in the striatum, and the majority of connections are established prior to neonatal life[17]. This fact suggests that transplantation of the dissociated VM from postnatal animals will induce substantial axotomy and subsequent cell death of DAergic neurons. However, this situation highly contrasts our observations of vast amounts of clearly expressed TH-ir cells after 8DIV.

The lack of cell death can be explained in several ways. First of all, the development of the striatal DAergic innervations continues throughout the third postnatal week[17], suggesting that several neurons have not yet formed axonal projections in the striatum at the time of explanation. This is further supported by the fact that postnatal VM slices cocultivated with the striatum establish TH-ir nigrostriatal projections *in vitro*[18]. Hence, a vast amount of the neurons we observe are possibly not subjected to axotomy. Second, neurons with established connections do not necessarily die following axotomization in organotypic slice cultures. As demonstrated by Tønder and colleagues[19], neonatal hippocampal pyramidal cells that have already projected commissural axons in the contralateral hippocampua survive subsequent intracerebral grafting and explantation as slice cultures, as shown by retrograde labeling with granular blue. Thus, a proportion of the observed neurons possibly originate from the axotomized neurons. Finally, it has been demonstrated that TH-ir neurons located in the aqueduct of the VM still show mitosis during the first

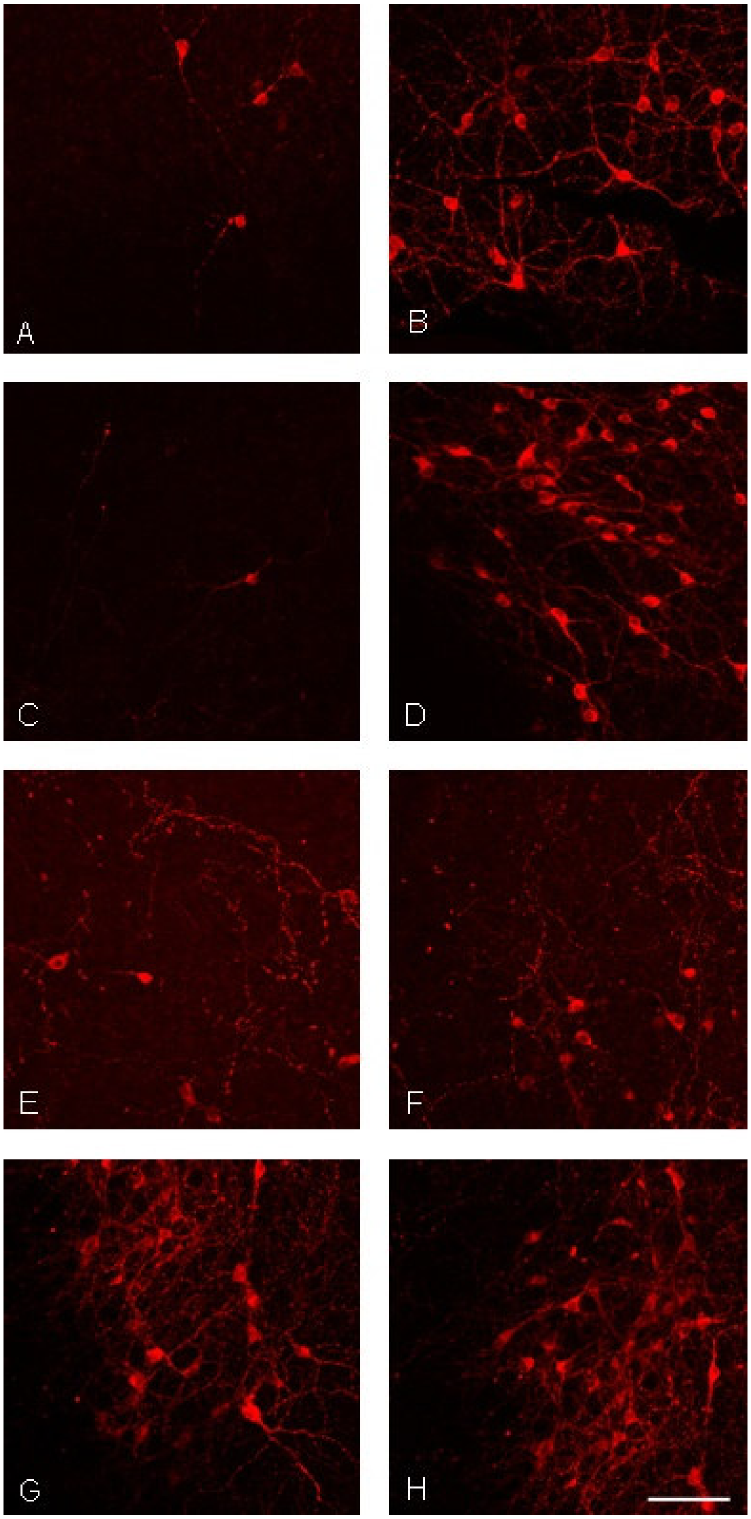

**FIGURE 5.** Comparison of different techniques and concentrations of 6-OHDA in slice cultures of SN. Loss of TH-ir cells is prominent in the hemisphere exposed to 200 μ*M* 6-OHDA (A). The untreated hemisphere remains unaffected (B), when compared to control cultures (G,H). Higher concentration (1000 μ*M*) of 6-OHDA results in almost complete cell death in the treated hemisphere (C). When the slice was exposed to a toxic bath of 6-OHDA (200 μ*M*), both hemispheres were equally affected (E,F) and made us unable to compare the cell death within the same slice. Scale bar; 50 μm.

postnatal week, suggesting that axotomized DAergic cells might be replaced by newly formed or inwardly generated neurons[20]. Taken together, early postnatal animals are thought to represent a compromise between established cytoarchitecture and axotomy.

Despite several advantages associated with organotypic cultures, the method is limited by complexity and tissue vulnerability. Such factors may include the dissection and the chopping procedure, environmental changes such as subtle variations in room temperature, cultivation factors such as small alterations in the media composition due to serum variation, and experimental procedures that involve removal from the incubator. All these factors influence the survival rate of the cultures and may induce considerable variation among the wells both within and between batches. These facts imply that there is a substantial need for internal controls when slices are treated with experimental factors, such as 6-OHDA, in order to attribute changes in the independent variable to treatment rather than changes induced by the organotypic model *per se*.

By using a unilateral toxic application technique in organotypic cultures, we introduce a simple method for generation of internal controls. This refined method eliminates certain confounding variables, as the untreated hemisphere serves as a direct control for the damages induced by the toxin. As mentioned, such confounders might be introduced by the organotypic model, but is also a result of variations in cell number along the rostrocaudal axis, both of which will induce considerable variability among slices. Accordingly, several parameters affect the number of DAergic cells by varying systematically or nonsystematically among the slices, and may therefore create a biased picture of the effect of degeneration in a toxic bath model. In contrast, the unilateral exposure model provides a reliable means for a direct comparison between the two sides, as variations can be directly attributed to the actions of the toxin. The statistical analysis clearly confirms the reliability of the unilateral method, as the effects of 6-OHDA on TH-ir cell counts result in a highly significant value ($p < 2 * 10^{-16}$). This indicates that the internal control provides a means for precise and reproducible assessments: By applying a relatively low concentration of 6-OHDA (200 μ*M*) in one hemisphere, one can expect approximately 80% cell death as compared to the opposite control hemisphere. Despite the clear significance, some variance exists among replications. This can be attributed to the complexity of the method. For instance, a slight angle during the chopping procedure may result in minor variations in TH-ir cell number in the two hemispheres prior to toxic application, or the toxic compound may affect the control hemisphere if the electrode is handled unsteadily.

Finally, the refined method closely resembles *in vivo* models where toxins are stereotactically and unilaterally injected into the striatum or the SN to produce lesions at specific sites, thus increasing the representativeness of the model.

All together, these advantages imply that organotypic cultures have increased their validity as a model for PD, and may therefore be used as a solid platform for studies of cytoprotective treatment. For instance, neurotrophic factors comprise a promising treatment for PD, but are restricted by contradicting results in regard to their efficiency and function. Using our refined version of organotypic cultures as a point of departure, one can possibly reveal some of these issues with a higher degree of validity.

### ACKNOWLEDGMENTS

We thank Øyvind Hvalby and Vidar Jensen in the Department of Physiology for their assistance in making the pipettes and the use of their facilities. Espen Petersheim is highly appreciated for editing the video.
